# Anterior variable-angle locked plating versus tension band wiring of simple and complex patella fractures – a biomechanical investigation

**DOI:** 10.1186/s12891-023-06394-x

**Published:** 2023-04-11

**Authors:** Karl Stoffel, Ivan Zderic, Torsten Pastor, William Woodburn, Richard Castle, Jessica Penman, Eladio Saura-Sanchez, Boyko Gueorguiev, Christoph Sommer

**Affiliations:** 1 University Hospital Basel, Petersgraben 4, Basel, 4031 Switzerland; 2 AO Research Institute Davos, Clavadelerstrasse 8, Davos, 7270 Switzerland; 3 Cantonal Hospital Lucerne, Spitalstrasse 16, Lucerne, 6000 Switzerland; 4 DePuy Synthes, Goshen Pkwy, West Chester, PA 1310, 19380 USA; 5 University Hospital of Elche, Carrer Almazara, 11, Elche, Alicante, 03203 Spain; 6 Cantonal Hospital Graubünden, Loestrasse 170, Chur, 7000 Switzerland

**Keywords:** Simple patella fracture, Complex patella fracture, Anterior variable-angle locked plating, Tension band wiring, Biomechanics

## Abstract

**Background:**

The aim of this study was to investigate the biomechanical performance of novel anterior variable-angle locking plates versus tension band wiring used for fixation of simple and complex patella fractures.

**Methods:**

Sixteen pairs of human cadaveric knees were used to simulate two-part simple transverse AO/OTA 34-C1 and five-part complex AO/OTA 34-C3 patella fractures. The complex fracture pattern was characterized with a medial and a lateral proximal fragment, together with an inferomedial, an inferolateral and an inferior fragment mimicking comminution around the distal patella pole. Eight pairs with simple fractures were split for fixation via either tension band wiring (TBW) through two parallel cannulated screws or anterior variable-angle locked plating, whereas other eight pairs with complex fractures were split for either TBW through two parallel cannulated screws plus circumferential cerclage wiring, or anterior variable-angle locked plating using a cortical caudo-cranial polar screw. Each specimen was tested over 5000 cycles with a range of motion from 90° flexion to full extension by pulling on the quadriceps tendon. Interfragmentary movements were captured by motion tracking.

**Results:**

For both fracture types, the longitudinal and shear articular displacements, measured between the proximal and distal fragments at the central patella aspect between 1000 and 5000 cycles, together with the relative rotations of these fragments around the mediolateral axis were all significantly smaller following anterior variable-angle locked plating versus TBW, p ≤ 0.01.

**Conclusions:**

From a biomechanical perspective, anterior locked plating of both simple and complex patella fractures resulted in less interfragmentary displacement under extended cyclic loading.

## Background

Fractures of the patella account for 0.5–1.5% of all fractures occurring in the human skeleton [1], with an increase in incidence of approximately 40% observed between the 1950s and 2011 [2]. Among the different types of patella fractures, the comminuted type AO/OTA 34-C3 is the most common, followed by simple transverse fractures type AO/OTA 34-C1 [3]. The injury mechanism in the majority of cases is a combination of direct trauma on the anterior patella surface with an indirect eccentric force exerted by contracted quadriceps muscle [4]. Displaced simple or comminuted patella fracture fragments associated with a disrupted extensor mechanism qualify for surgical treatment [1]. Thereby, anatomic reduction with reestablishment and perseverance of a smooth articular surface is given high importance to prevent posttraumatic osteoarthritis [5, 6], the latter being prone to develop due to high patellofemoral joint forces [7, 8].

Transverse patella fractures are commonly fixed via tension band wiring (TBW) in all its modifications [9, 10], most frequently using two parallel longitudinal Kirschner (K-) wires or cannulated screws inserted perpendicularly to the fracture plane together with a cerclage wire placed anteriorly in a figure-of-eight pattern around their extrusion [11–13]. Alternative fixation methods, such as using staples or intraosseous nailing, have been reported but are rather less common [8, 14, 15].

The lack of congruity is especially pronounced in comminuted patella fractures [16]. Their surgical treatment can be performed via TBW, usually requiring additional circumferential cerclage wiring [4, 17]. On the other hand, the maintenance of congruity and neutralization of tensile forces are believed to be more efficient when using low-profile locking plates [18, 19]. Different techniques have been described, comprising anterior plating with monocortical screws [20] and various lateral plating techniques.

**Fig. 1:**
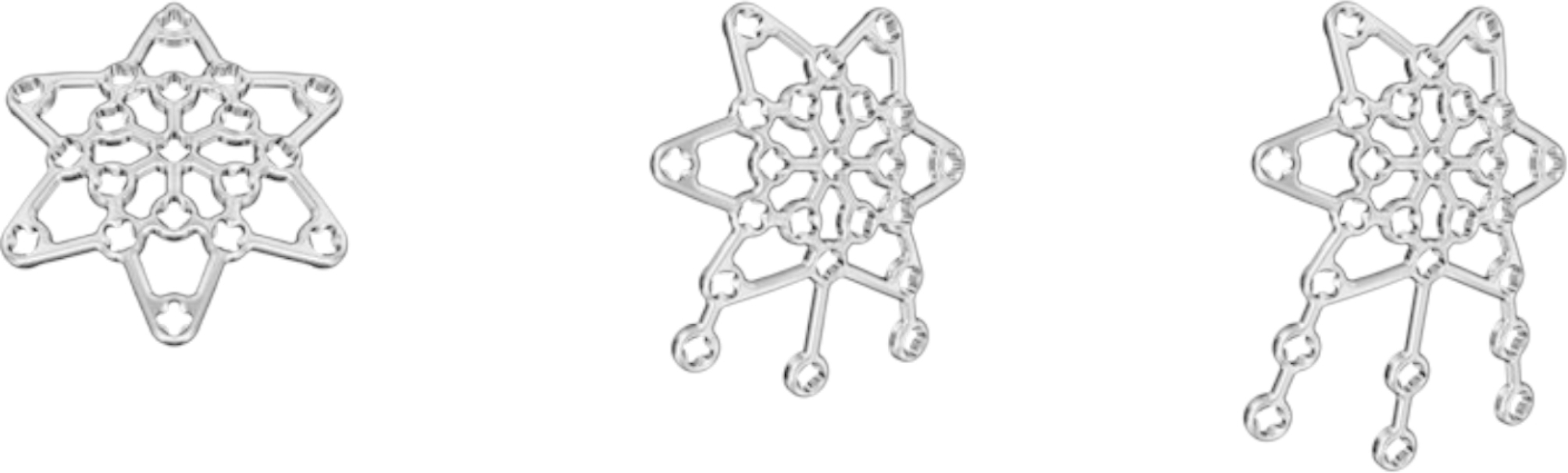
Core (left), three-hole (middle) and six-hole (right) standard Variable-Angle Locking Anterior Patella Plates 2.7 designed for treatment of simple and complex patella fractures

Recently, anterior variable-angle (VA) locking plates were developed for specific treatment of both simple and comminuted patella fractures, establishing an armamentarium forimplementation of multiplanar fixation (Fig. 1). The plates, made of medical grade stainless steel (316 LVM) or Titanium (cp-Ti), are provided in two sizes with three different configurations each. The configurations vary in the three leg branches for capturing of the distal patella pole, namely core (without legs), three-hole, and six-hole. The radially arranged six arms and the distally extending three legs of the plate are designed to be bent, allowing for best-possible patient-specific fit on the corresponding patella surface fragment. The legs allow bicortical polar (apex to base) screws placement for interfragmentary fixation. Each plate hole can accept 2.4 and 2.7 mm VA locking or cortex screws [21].

The aim of this study was to investigate the biomechanical performance of the recently developed anterior VA locking plates versus TBW used for fixation of simple and complex patella fractures. It was hypothesized that plating would demonstrate less interfragmentary movements under dynamic loading.

## Methods

### Specimens and preparation

Sixteen pairs of fresh-frozen (-20 °C) human cadaveric knees from eight female and eight male donors (mean age 78 years, range 67–93 years) were used. The inclusion criteria were restricted to donors’ body height ranging between 165 and 180 cm, and equal gender distribution. No limitations in terms of age or similar applied to specimens’ collection. All donors gave informed consent within the donation of anatomical gift statement during their lifetime. All experiments were carried out under the relevant guidelines and regulations. Additionally, internal review boards at Science Care (Phoenix, AZ, USA) and the AO Research Institute Davos approved the project (PP2115, 6 February 2018). The knees were thawed at room temperature for 24 h prior to preparation and biomechanical testing. Each femur and tibia were transected at a distance of 15 and 25 cm from the knee joint, respectively.

The patellae of all knees were assessed for bone mineral density (BMD) within their trabecular region via computed tomography (CT) scanning (Revolution EVO, General Electric Healthcare, Buckinghamshire, UK) using a phantom (BDC-6, QRM GmbH, Möhrendorf, Germany) and subsequent image analysis (Amira, v.6.0, Thermo Fisher Scientific, Waltham, MA, USA) with segmentation between 150 and 450 mgHA/cm^3^.

**Fig. 2:**
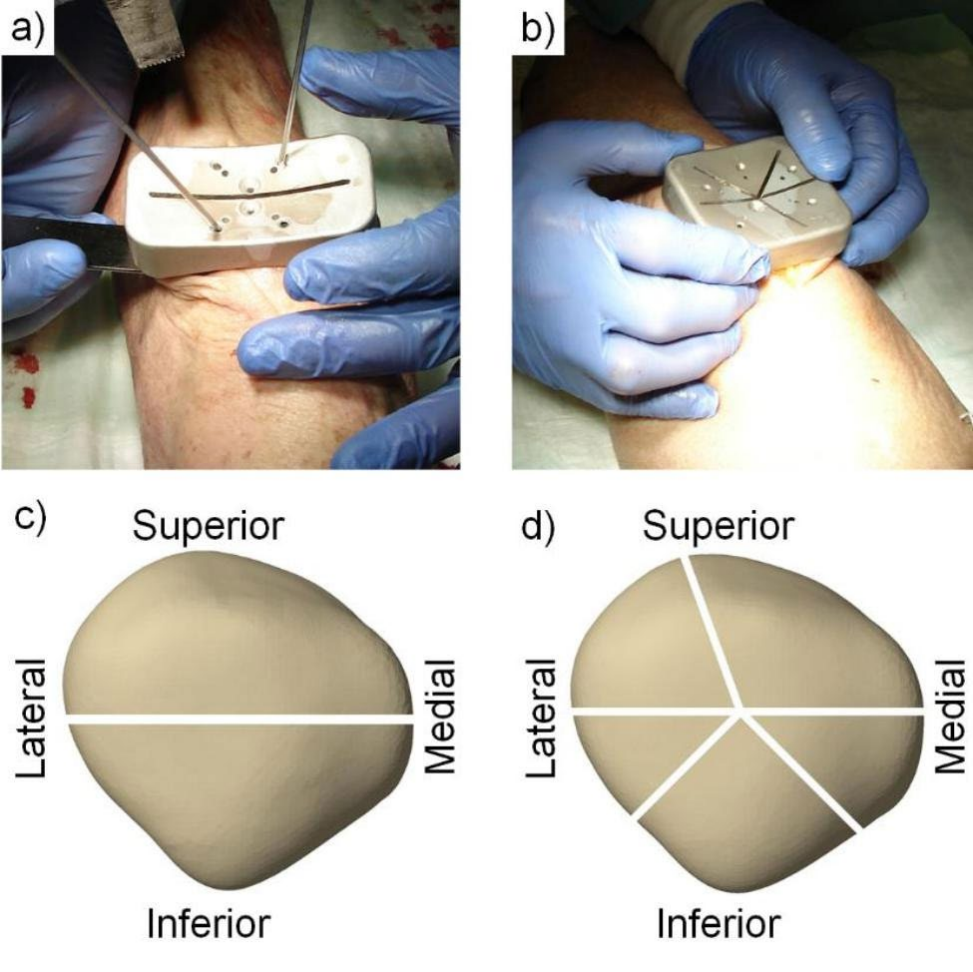
Photographs of cutting guides positioned on specimens for creation of a simple **(a)** or a complex **(b)** fracture. Schematic views of the simple **(c)** and complex **(d)** fracture models

Based on BMD, the knees were randomized to two clusters consisting of eight pairs each, such that the BMD values were homogeneously distributed between the two clusters (p = 0.497). The specimens in one of the clusters were assigned for creation of a simple two-part transverse patella fracture type AO/OTA 34-C1, whereas the knees in the other cluster were assigned for simulation of a complex comminuted five-part patella fracture type AO/OTA 34-C3. Further, two groups with four right and four left specimens each (n = 8) were created within each cluster by splitting of its pairs. The sample size was calculated based on a priori power analysis for statistical power of 0.8 at a level of significance 0.05 under the assumption that the standard deviation (SD) within each group is not bigger than 80% of the minimum difference between the mean values among the groups. A mid-axial longitudinal approach was used to expose the anterior patella surface. A horizontal cutting line was marked centrally after identification of the proximal and distal poles. Fracture creation was performed via osteotomies using custom cutting guides attached on the anterior patella surface (Fig. 2a-b). Each cutting guide was aligned with the corresponding horizontal mark. The simple fracture pattern was set by a single cut through the transverse slot. The complex fracture pattern was characterized with a smaller medial and a larger lateral proximal fragment, together with an inferomedial, an inferolateral and an inferior (central distal) fragment mimicking comminution around the distal patella pole. The corresponding slots of the cutting guide were designed radially, crossing each other at one central point. All fragments were fully separated from each other during osteotomizing (Fig. 2c-d). The extensor retinaculum was divided in line with each transverse cut. A lateral arthrotomy was performed for anatomical fracture reduction, provisionally fixed with pointed forceps and temporary K-wires. A smooth articular surface was confirmed by palpatory inspection.

The two paired groups with simple patella fractures were assigned for fixation with either TBW through two parallel cannulated screws or using an anterior VA locking core plate. The TBW procedure commenced with retrograde insertion of two parallel 1.6 mm K-wires across the fracture gap, followed by their over-drilling using a 3.2 mm cannulated bit. A short-threaded 4.5 mm cannulated screw was inserted over each K-wire in retrograde fashion. The length of each screw was measured individually for each specimen, considering that the screw tip remains completely intraosseous. Two sections of a 1.25 mm cerclage wire were inserted through each cannula of the screws, crossed, and positioned in a figure-of-eight fashion.

The anterior plating of the simple fractures started with selection of the plate size individually for a best-possible match to the specimen’s patella. The next step comprised plate positioning and its provisional attachment with compression K-wires. Care was taken to align the two outer star aspects of the plate with the longitudinal patella midline. When necessary, the two corresponding proximal and distal arms were individually bent to ensure optimal fit to the patella surface using dedicated bending instruments. Pilot holes were pre-drilled with 2.0 mm drill bits using VA drill guides, allowing to set appropriate drill hole direction with a maximum deviation from the nominal axis of the plate hole up to 15°. Following, 2.7 mm VA locking screws were inserted in the pilot holes and locked at 1.2 Nm in unicortical fashion. The proximal and distal three holes of the plate arms were occupied with screws together with the two inner plate body holes, resulting in a total of ten screws used for plate fixation.

The other two paired groups with complex fractures were assigned for fixation with either TBW through two parallel cannulated screws plus circumferential cerclage wiring or using an anterior VA locking three-hole plate.

The TBW of the complex fractures resembled the one applied for simple fractures. Care was taken that all fragments were traversed by the screw trajectories. An additional 1.25 mm cerclage wire was circumferentially placed around the patellar rim.

The anterior plating of the complex fractures commenced with placement and orientation of the plate, conducted as needed to best fixate the fragments based on specimen’s anatomy. Care was taken for each fragment (1) to receive a screw through at least one of the plate arm holes, and (2) to interact with at least two screws. Plate arms and legs were bent, the latter to the extent so that each of them embraced one of the three distal fragments basket-wise. Screw placement in the proximal and distal three holes of the arms, and where applicable in the two inner plate body holes was ensured as for the anterior plating of simple fractures. Moreover, the three distal fragments were fixed through the leg holes using one cortical polar screw for the inferior fragment and two locking screws for the inferomedial and inferolateral fragments, allvectored caudo-cranially in the frontal plane and transfixing additionally the transverse fracture line in bicortical fashion.

**Fig. 3:**
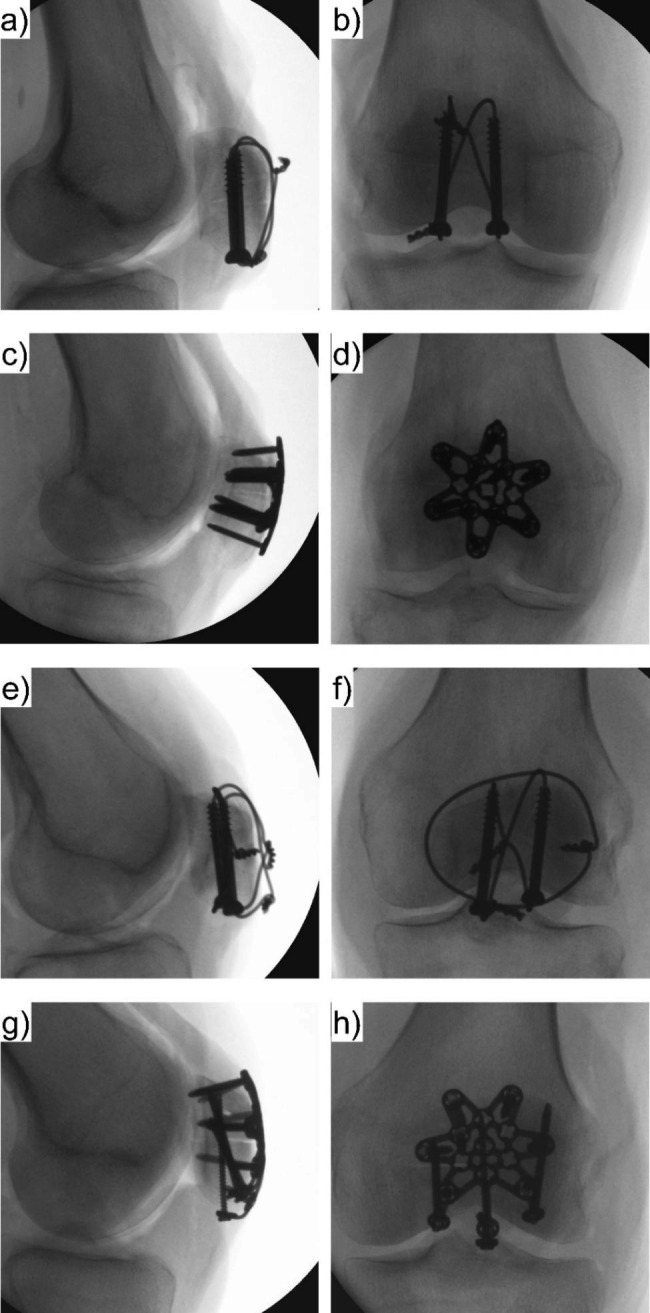
Exemplified medio-lateral **(a,c,e,g)** and antero-posterior **(b,d,f,h)** radiographs of specimens with simple **(a-d)** and complex **(e-h)** fractures treated by TBW **(a,b,e,f)** or anterior locked plating **(c,d,g,h)**

All used implants were made of stainless steel and provided by the same manufacturer (DePuy Synthes, Zuchwil, Switzerland). Two experienced surgeons performed all instrumentations under fluoroscopic control (Fig. 3) ensuring consistency of the procedures. Skin and subcutaneous tissue were completely excised after instrumentation. The fibula and all surrounding muscles were removed, with exception of the extensor mechanism, ligaments, and joint capsule of the knee that were preserved. The proximal 6 cm of the femur and the distal 6 cm of the tibia were embedded in a polymethylmethacrylate (PMMA, Suter Kunststoffe AG, Fraubrunnen, Switzerland) socket. A threaded steel rod was secured in the intramedullary canal of the tibia during embedding, allowing to attach a disc of 3.1 kg weight to it at a distance of 25 cm from the knee joint to mimic lower limb weight [7, 8, 22, 23]. Retro-reflective marker sets were attached to the tibia, as well as either to the proximal and distal patella fragments in case of a simple fracture, or to the medial proximal and central distal fragments in case of a complex fracture for motion tracking.

### Biomechanical testing

**Fig. 4:**
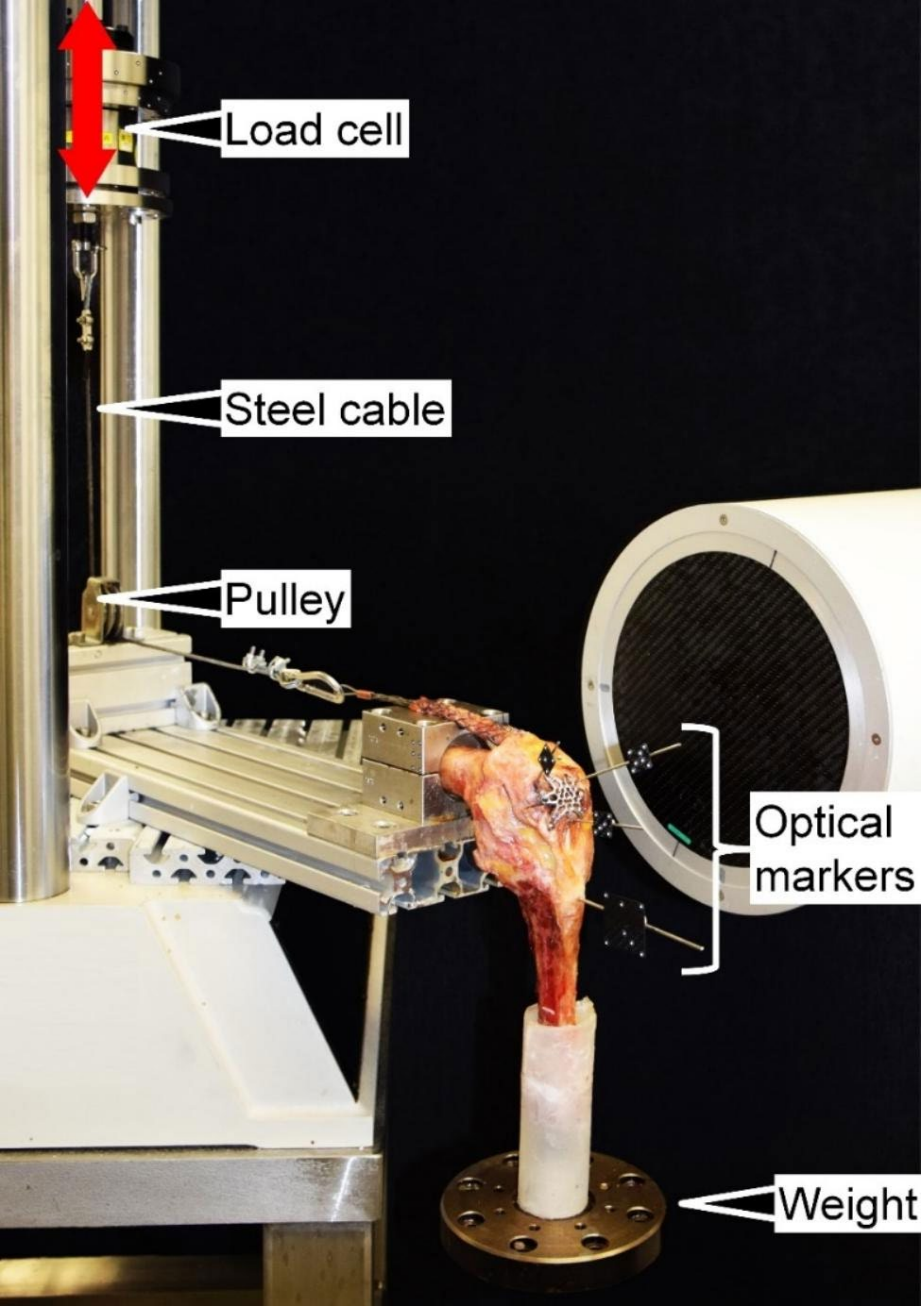
Setup with a specimen implanted with a three-hole standard Variable-Angle Locking Anterior Patella Plate 2.7, equipped with markers for motion tracking and mounted for biomechanical testing. Vertical double-arrow indicates movement direction of the machine transducer

Biomechanical testing was performed on a servo-hydraulic material testing machine (Bionix 858.20, MTS Systems Corp., Eden Prairie, MN, USA) – equipped with a 4 kN load cell (HUPPERT 6, HUPPERT GmbH, Herrenberg, Germany) and operating at class 1 accuracy – adopting a test setup and a loading protocol from previous studies (Fig. 4) [14, 16]. The proximal part of each femur was mounted horizontally on the machine base. Pulling force was transmitted to the quadriceps tendon via a steel cable directed via a pulley to the machine actuator.

Each specimen underwent cyclic loading at 1/6 Hz over 5000 cycles mimicking knee movements of a sitting patient between 90° flexion and full knee extension. Starting from a preload of 20 N with the knee flexed at 90°, the quadriceps tendon was pulled during each cycle until full knee extension and then returned to 90° flexion, applying a bell-shaped loading profile with 300 N peak tensile force.

### Data acquisition and analysis

Machine data in terms of actuator displacement and pulling force were collected from the test system controllers at 128 Hz. In addition, two optical cameras (Aramis SRX, GOM GmbH, Braunschweig, Germany) continuously recorded the marker positions for motion tracking, operating at resolution of 12 megapixel and a maximum acceptance error of 0.004–0.02 mm [24, 25]. Interfragmentary movements were evaluated at the initial stage after 50 loading cycles and then after 1000, 2000, 3000, 4000 and 5000 cycles as follows. Fracture site displacement along the tibia axis – defined as longitudinal displacement – and perpendicular to it in antero-posterior direction – defined as shear displacement – was calculated at the most articular margin of the horizontal osteotomy plane located centrally in medio-lateral direction. For that purpose, the location of the articular margin was registered prior to test start by using a touch probe and assigned virtually in a rigid-body constellation (1) once to the proximal and once to the distal fragment in case of a simple fracture, or (2) once to the medial proximal and once to the central distal fragment in case of a complex fracture. Based on this, the relative displacement between these two virtually registered margins was calculated along and perpendicular to the tibia axis, the latter in antero-posterior direction. Further, interfragmentary rotation around the mediolateral axis was evaluated. All three metrics were derived at 90° knee flexion and calculated between the proximal and distal fragments for simple fractures, and between the medial proximal and central distal fragments for complex fractures.

Statistical analysis was performed using SPSS software package (IBM SPSS Statistics 27, IBM, Armonk, NY, USA). Normality of data distribution within each fracture model and fixation technique was screened and proved with Shapiro-Wilk test, following One-Way Analysis of Variance (ANOVA) to confirm appropriate randomization of the specimens based on BMD. Paired-Samples T-test and General Linear Model Repeated Measures test were applied for each separate cluster of specimens to identify significant differences between the corresponding fixation techniques. Level of significance was set to 0.05 for all statistical tests.

## Results

BMD (mgHA/cm^3^) was 207.5 (41.0) (mean (standard deviation, SD)) for anterior plating and 210.6 (34.7) for TBW in the cluster of specimens with a simple fracture, and 214.5 (44.2) for anterior plating and 219.9 (36.8) for TBW in the cluster of specimens with a complex fracture, demonstrating a homogeneous distribution among the groups (p = 0.251).

The results for the three outcomes characterizing interfragmentary movements after 50 loading cycles and summarized in Table 1, feature anterior plating as having (1) significantly smaller rotation around the medio-lateral axis for both fracture types (p ≤ 0.013) and (2) significantly smaller shear displacement for the complex fractures (p = 0.002) versus TBW, with no further significant differences between the paired groups (p ≥ 0.102).

**Table 1:** Outcomes after 50 loading cycles for each separate fixation technique (anterior plating (Plate) or TBW) and fracture model (simple or complex fracture) presented in terms of mean value and standard deviation, together with the corresponding p-values from the statistical comparisons between the paired groups

Outcome	Plate	TBW	P-value
**Simple fracture**			
Longitudinal displacement (mm)	0.008 (0.013)	0.182 (0.271)	0.169
Shear displacement (mm)	0.003 (0.006)	0.051 (0.133)	0.403
Interfragmentary rotation (°)	0.091 (0.115)	0.728 (0.324)	0.002
**Complex fracture**			
Longitudinal displacement (mm)	0.001 (0.004)	0.116 (0.144)	0.102
Shear displacement (mm)	0.021 (0.087)	0.273 (0.214)	0.002
Interfragmentary rotation (°)	0.076 (0.141)	1.602 (1.084)	0.013

**Fig. 5:**
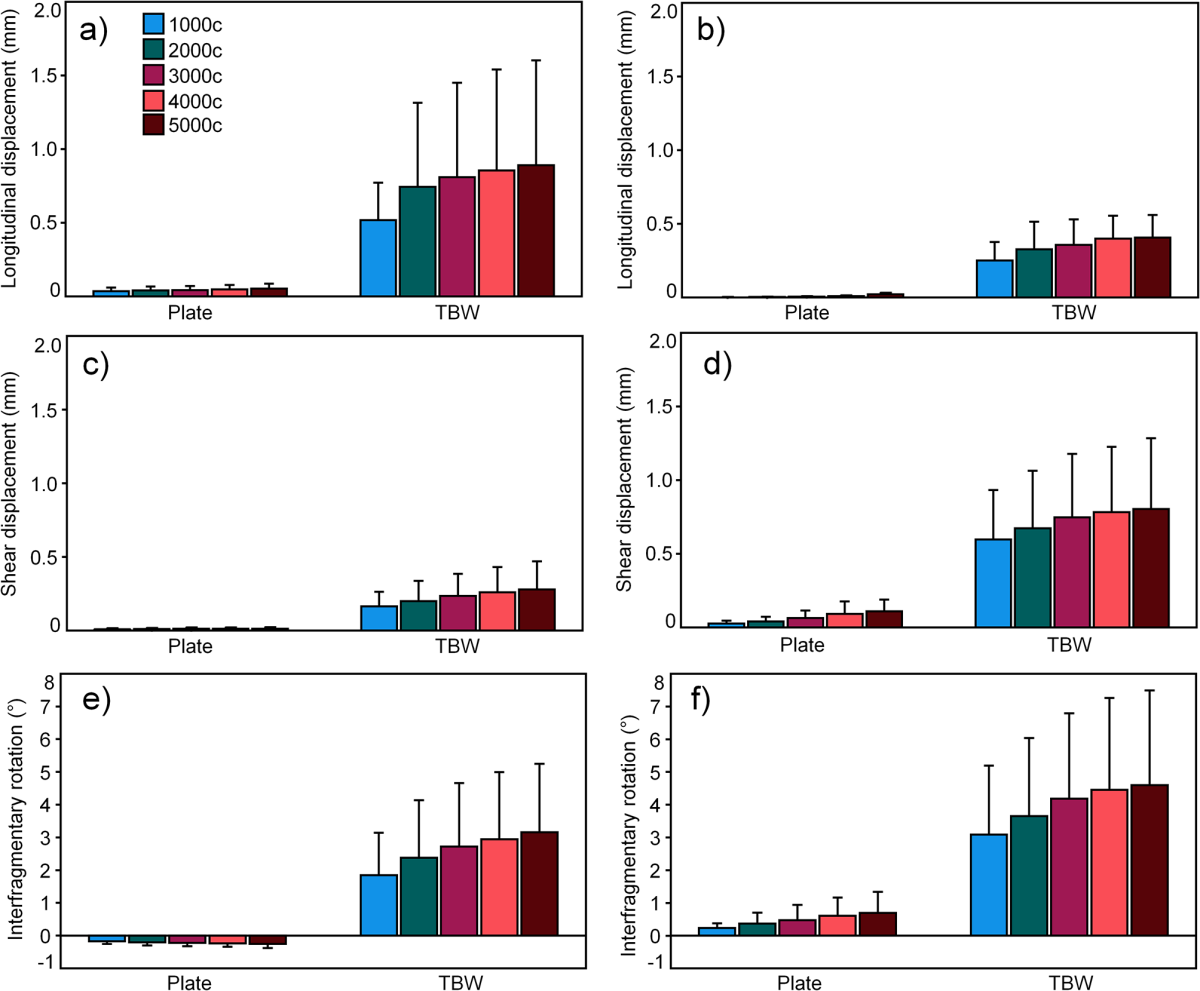
Longitudinal **(a,b)** and shear **(c,d)** articular displacement at the central aspect of the patella, and interfragmentary rotation around the medio-lateral axis **(e,f)** in terms of mean value and standard deviation, measured between the corresponding proximal and distal patella fragments after 1000, 2000, 3000, 4000 and 5000 test cycles and featuring simple **(a,c,e)** and complex **(b,d,f)** fractures fixed by either anterior variable-angle locked plating (Plate) or TBW.

For each separate fracture type, the outcome measures for all three investigated metrics, evaluated after 1000, 2000, 3000, 4000, and 5000 loading cycles, were significantly smaller following anterior plating versus TBW (p ≤ 0.010, Fig. 5; Table 2). For both fracture types, anterior plating was associated with a significant increase of all outcome measures over time (p ≤ 0.016). In contrast, the outcome measures for TBW increased significantly only for shear displacement and interfragmentary rotation within the simple fracture type, and for longitudinal and shear displacement within the complex fracture type (p ≤ 0.041), whereas they remained comparable for longitudinal displacement within the simple fracture and interfragmentary rotation within the complex fracture type (p ≥ 0.101). BMD revealed no significant influence on the results when considered as a covariate (p ≥ 0.084) (Table 2).

**Table 2:** P-values (1) for outcomes’ progressions over time after 1000, 2000, 3000, 4000, and 5000 loading cycles (Increase over cycles) considering each separate fixation technique (Plate or TBW), (2) from outcomes’ comparisons between fixation techniques (Plate versus TBW), and (3) for the effect of BMD on the outcome measures for each separate fixation technique (Plate or TBW). All values are presented separately for simple and complex fractures

Outcome	Increase over cycles		Plate versus TBW	Effect of BMD	
	Plate	TBW		Plate	TBW
**Simple fracture**					
Longitudinal displacement	0.009	0.105	0.010	0.219	0.569
Shear displacement	0.010	0.041	0.004	0.503	0.756
Interfragmentary rotation	0.016	0.015	0.003	0.084	0.245
**Complex fracture**					
Longitudinal displacement	0.005	0.020	< 0.001	0.970	0.106
Shear displacement	0.004	0.025	0.002	0.797	0.913
Interfragmentary rotation	0.005	0.101	0.006	0.835	0.357

**Fig. 6:**
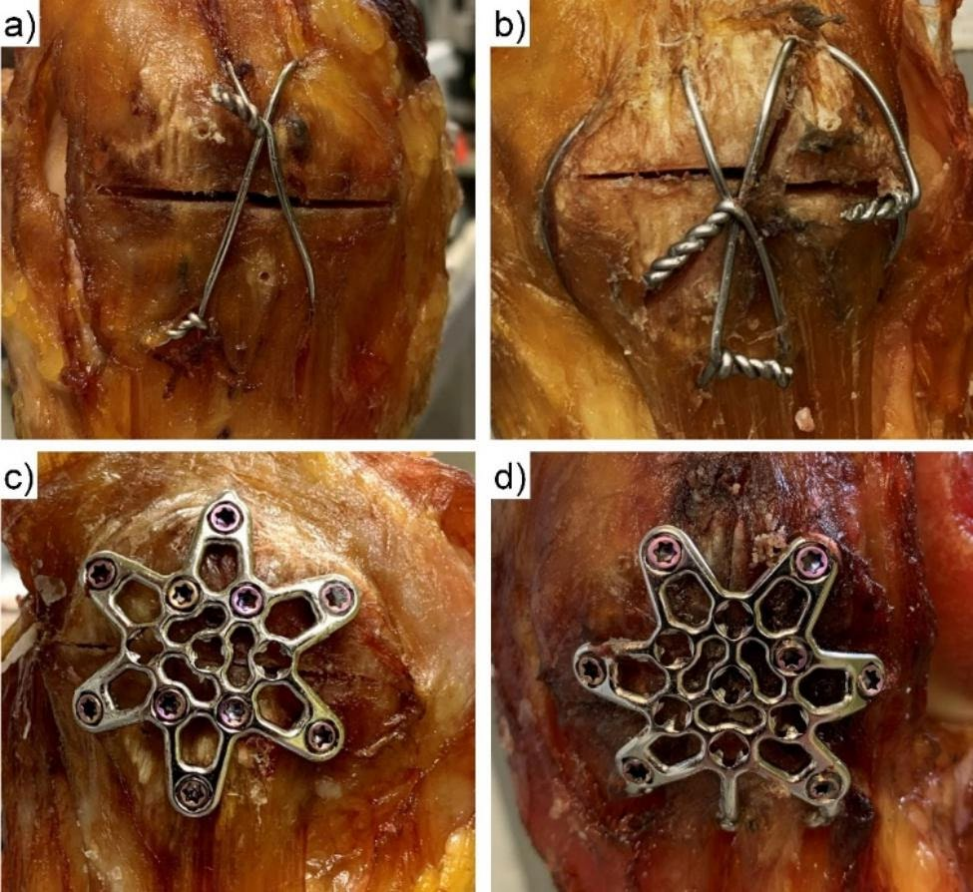
Exemplified post-test photographs of specimens with simple **(a,c)** or complex **(b,d)** fractures treated by TBW **(a,b)** or anterior variable-angle locked plating **(c,d)**

All cyclic tests were completed without catastrophic implant failure in any of the specimens (Fig. 6).

## Discussion

The current study investigated biomechanically the performance of novel VA locking plates versus TBW with cannulated screws in a human cadaveric model with simulated simple and complex patella fractures. For both fracture types, the anterior locked plating demonstrated significantly smaller interfragmentary movements with greater homogeneity.

The overall combined longitudinal and shear displacements were of similar magnitude in both simple and complex fracture models treated with the same fixation technique. Further, the transverse fracture line, being the most critical aspect for patella fixation even in complex fractures, was associated with comparable interfragmentary rotation around the medio-lateral axis in both fracture models.

Interestingly, a significant increase of interfragmentary movements within the course of cyclic testing was observed for anterior plating, whereas for TBW it remained partially non-significant. This is another non-intuitive result that can be explained by the fact that instability settled in at the early stage of cyclic testing for TBW, and then remained at that relatively high level. On the other hand, the intefragmentary movements increased over time for anterior plating, but remained at a very low level.

The visual inspection of the tested specimens post testing revealed distinct gap opening at the transverse fracture line of the specimens fixed by TBW, whereas those treated with anterior plating kept all fracture sites closed to each other. For TBW, the ability to keep the fracture gap closed relies on both the friction between the winded cerclage wires and their proximity to the bone, i.e., on the amount of soft tissue between cerclage and bone. Both factors may not be reliable enough for satisfactory strength of fixation. Whereas a friction coefficient, lowered by interaction with surrounding soft tissue, may lead to unwinding of the cerclage, the soft tissue lying between bone and cerclage may impede anatomical clinging of the cerclage to the bone. This does not apply for anterior locked plating, where fixation is achieved and maintained via angular stable screw fixation. Thereby, the locking mechanism is predominantly stressed in cantilever bending and shearing at the screw head for the antero-posterior screws, while the polar screws undergo axial pullout forces combined with bending moments. However, in the current testing series no breakage of plate fixation screws was observed.

The distally comminuted patella zone is challenging and requires manual dexterity to address each of the small fragments. This circumstance may be alleviated using the caudally emerging legs of the anterior plates that can be bent basket-like around the distal patella pole to capture all fragments in the comminuted zone. Moreover, this plate feature presents a further cornerstone for improved fixation stability by allowing insertion of polar screws from caudal to cranial in the frontal plane for fracture fixation in two orthogonal planes.

Whereas previous studies performed extensive biomechanical investigations on simple patella fracture plating [22, 26–30], the assessment of its efficacy for fixation of comminuted patella fractures is sparce [16, 31, 32]. The current study best compares to the one published by Kfuri et al. [16] in threefold manner, namely regarding the fracture model, the applied fixation techniques, and the type of investigation as follows. In the previous study the authors investigated for the first time the biomechanical competence of techniques used for fixation of comminuted patella fractures. A six-part unstable fracture with lack of circumferential contact between the anterior cortices and with a coronal split of the distal fragments was simulated. Fixation techniques comprised TBW using K-wires and additional screws, as well as anterior and lateral locked mesh plating with off-label application. Biomechanical testing was identical to the current study. The authors concluded that, providing superior stability, anterior plating is a valid alternative to TBW for patella fracture treatment. This statement is true for the present results too, although the fracture model and type of used implants differed in detail.

Several clinical trials, case reports and surgical techniques have been published, using a variety of locked plating techniques for treatment of complex patella fractures [19, 20, 33–42]. Despite the promising results, and although seldom, there are still postoperative complications that may arise following plating of patella fractures, including wound infection, fracture displacement/loss of reduction [19], hardware irritation [37], limited mobility up to arthrofibrosis [38, 42], or even implant failure [41]. It has to be considered that different plate designs may affect the postoperative outcomes. The plate presented in this study underwent no clinical trials yet, and therefore, no data can be reported in this regard. Potential complications may arise when implant removal is required, depending of bone-implant integration. In addition, care must be taken to avoid penetration of anteriorly placed screws to the articular surface. Whereas limitations following patella plating are low, there are still proponents successfully advocating the continued use of TBW in all its modifications, even for fixation of difficult patella fractures with comminution [43–48]. One reason for this are the lower costs associated with TBW. Furthermore, TBW is a versatile technique and can be modified to purpose-fit the present need. Experienced and manually skilled surgeons may well take advantage of this versatility to achieve sufficient fixation stability, although TBW may be less forgiving. In addition, some clinical trials demonstrated no evidence on the effects of different surgical interventions [43, 49]. Finally, fixation failures may be a result of patient non-compliance instead of the TBW technique itself [43, 46]. On the other hand, poor mid-term functional results have been witnessed following TBW [1, 15, 50–52].

This study has some limitations inherent to all human cadaveric investigations using a limited number of specimens. Moreover, within the knee specimens with comminuted fractures not all fragments could be monitored via motion tracking due to setup-related technical reasons. However, the most important anticipated relative movements were identified and accordingly tracked. Next, and inherent with the used comminuted fracture model, all distal fragments could be addressed with the plate screws, which may not be the case for other types of comminuted fractures. In this regard, an anatomical study applying different fracture models may assess the universality of the current plate design. Finally, given the nature of a biomechanical study, evaluation of potential postoperative complications was not feasible, which warrants further clinical investigations.

Methodological strengths of this study include the use of an accurate motion tracking system, allowing precise investigation of interfragmentary movements. Moreover, the statistical power deemed high enough as significant differences between the groups were demonstrated for the most relevant parameters. Finally, a matched paired design was used for each fracture model, ensuring comparable conditions between the tested fixation techniques.

Future studies shall focus on the biomechanical comparison between the different plating systems available on the market to assess their strengths and weaknesses. Such comparisons serve as groundbreaking for further implant design optimizations.

## Conclusions

From a biomechanical perspective, anterior locked plating of both simple and complex patella fractures resulted in less interfragmentary displacement under extended cyclic loading. Further research is necessary to determine the clinical relevance of these findings.

## Data Availability

The datasets used and/or analysed during the current study are available from the corresponding author on reasonable request.

## List of abbreviations

TBW: Tension Band Wiring
K-wire: Kirschner wire
VA: variable angle
BMD: bone mineral density
CT: computed tomography
PMMA: polymethylmethacrylate
SD: standard deviation
ANOVA: analysis of variance
